# Association of Glucose-Dependent Insulinotropic Polypeptide Receptor Polymorphisms rs3848460 and rs3895874 with the Risk of Gestational Diabetes Mellitus

**DOI:** 10.2174/0118715303367483250728105845

**Published:** 2025-08-05

**Authors:** Gyan Watson Ray, Hengli Zhang, Taotao Shao, Taili Yang, Yue Wei, Mianqin Li, Xiaoqun Che, Qiaoli Zeng, Runmin Guo

**Affiliations:** 1 Department of Internal Medicine, Shunde Women and Children's Hospital (Maternity and Child Healthcare Hospital of Shunde Foshan), Guangdong Medical University, Foshan, Guangdong, China;; 2 Key Laboratory of Research in Maternal and Child Medicine and Birth Defects, Guangdong Medical University, Foshan, Guangdong, China;; 3 Maternal and Child Research Institute, Shunde Women and Children's Hospital (Maternity and Child Healthcare Hospital of Shunde Foshan), Guangdong Medical University, Foshan, Guangdong, China;; 4 Department of Ultrasound, Shunde Women and Children's Hospital (Maternity and Child Healthcare Hospital of Shunde Foshan), Guangdong Medical University, Foshan, Guangdong, China;; 5 Department of Obstetrics, Shunde Women and Children's Hospital (Maternity and Child Healthcare Hospital of Shunde Foshan), Guangdong Medical University, Foshan, Guangdong, China;; 6 *Reproductive Medicine Center, Shunde Women and Children's Hospital (Maternity and Child Healthcare Hospital of Shunde Foshan), Guangdong Medical University, Foshan, Guangdong, China*

**Keywords:** Case-control study, GIP, gestational diabetes mellitus, rs3848460, rs3895874, single-nucleotide polymorphisms

## Abstract

**Background:**

This study aimed to examine the relationship between glucose-dependent insulinotropic polypeptide (GIP) polymorphisms rs3848460 and rs3895874 and the development of gestational diabetes mellitus (GDM) in Asian women. GIP, a hormone that stimulates insulin secretion and inhibits glucagon release, can be impacted by genetic variations, resulting in a reduced insulin response and elevated blood sugar levels that contribute to the development of GDM. To the best of our knowledge, only a limited number of studies have been conducted on the association between GIP gene polymorphisms and GDM.

**Methods:**

The SNPscanTM genotyping assay was employed to genotype rs3848460 and rs3895874, with 502 control participants and 500 GDM patients selected for the study. ANOVA, T-test, chi-square test, logistic regression, and various other statistical tests were employed to investigate variations in genotypes and alleles and their associations with the risk of GDM.

**Results and Discussion:**

In this study, significant differences were found in pre-BMI, age, systolic blood pressure, diastolic blood pressure, and parity between GDM and healthy subjects (*P* < 0.05). In the codominant model, GIP rs3848460 showed a significant association with an increased risk of GDM after adjusting (GG *vs*. AA: OR = 1.717; 95% CI: 1.070-2.754; *P* = 0.025. The recessive model GG *vs*. AG+AA suggests an elevated risk of GDM (adjusted OR of 1.635), with *P* = 0.034. The GG genotype and G allele demonstrated a statistically significant increased risk with an adjusted OR of 1.760 (*P* = 0.026) and an adjusted OR of 1.250 (*P* = 0.029), respectively. The GG genotype and G allele were found to be associated with an increased risk of GDM in GIP rs3848460. Conversely, the risk of GDM was significantly lower in the dominant model (AA+GA *vs*. GG: OR = 0.605; 95% CI: 0.376-0.974; *P* = 0.039) for GIP rs3895874. The AA genotype and A allele were correlated with a decreased risk of GDM in GIP rs3895874 after adjustment with an OR of 0.803 (*P* = 0.032).

**Conclusion:**

GIP rs3848460 was found to be significantly associated with the risk of GDM. Conversely, the risk of GDM was significantly lower in rs3895874.

## 1 INTRODUCTION

The gastrointestinal tract, recognized as the largest endocrine organ in the human body, plays a critical role in hormone production, influencing energy expenditure and body weight through various signaling and sensing mechanisms [1]. Among the peptides released from the gut in response to dietary intake, glucose-dependent insulinotropic polypeptide (GIP) [1] is notable for its ability to enhance insulin secretion when blood sugar levels are high [2]. The production of GIP by islet cells and its physiological importance in insulin synthesis have been highlighted in several studies [3]. The influence of GIP on β-cell mass is particularly significant in understanding the pathophysiology of both type 1 diabetes (T1DM) and type 2 diabetes (T2DM) [4, 5]. While patients with type 2 diabetes exhibit impaired incretin function, this deficiency is generally viewed as a consequence of the diabetic state rather than its cause [6]. However, research [7] suggests that impaired incretin effect might be a critical factor in the onset of type 2 diabetes.

Gestational Diabetes Mellitus (GDM) presents a major health concern during pregnancy, affecting both maternal and fetal outcomes [8]. It represents the most frequent medical complication of pregnancy and is characterized by diabetes that first manifests during the gestational period without prior diagnosis of overt diabetes [9, 10]. Studies have indicated that fasting levels of GLP-1 are higher in patients with GDM compared to non-GDM pregnant women. However, the differences in GLP-1 levels reported in some studies are not statistically significant [11, 12].

The hormone GIP, secreted by enteroendocrine K cells, plays a central role in glucose metabolism and insulin secretion, functioning as a key incretin hormone. GIP, secreted by enteroendocrine K cells in the small intestine following a meal, binds to GIP receptors (GIPRs) in various tissues, including the brain, stomach, adipose tissue, and pancreatic islets, where it exerts diverse metabolic effects. This widespread receptor expression underlines the broad role of GIP signaling in managing conditions like type 2 diabetes, GDM, obesity, and insulin resistance. GIP stimulates insulin secretion, which may play a role in the development of GDM in pregnant women. The exact mechanism is currently under investigation, but it is believed to increase insulin resistance, ultimately leading to elevated blood sugar levels and a diagnosis of GDM.

Rodent studies have shown that GIP can modulate insulin secretion in response to glucose and influence beta-cell neogenesis, differentiation, and proliferation within the pancreas, collectively described as intrapancreatic effects [13-15]. Beyond its pancreatic effects, GIP also exhibits extrapancreatic influences, such as enhancing lipoprotein lipase activity in cultured 3T3-L1 adipocytes. Genetic studies have linked variations in GIPR to plasma glucose levels, incretin effects, and body mass index (BMI) across different populations [16-18]. Despite extensive investigation into the pathophysiology of GDM, the specific molecular mechanisms driving its development remain unclear. There is a significant research gap in understanding the function and association of GIP polymorphism in GDM. Few studies have shed light on the unique role of GIP in GDM, which differs from its roles in type 1 and type 2 diabetes. Lower levels of GIP have been associated with an increased risk of developing GDM, suggesting a potential protective role in regulating blood sugar levels during pregnancy [19]. Conversely, the role of GIP in type 1 and type 2 diabetes is more complex. While some studies suggest elevated GIP levels in individuals with type 2 diabetes [19, 20], conflicting findings have also been reported [11]. To address this knowledge gap, our study focuses on exploring the association between GIP and GDM. To the best of our knowledge, our research represents the first investigation into the association between the GIP polymorphisms rs3848460 and rs3895874 and susceptibility to GDM.

The objective of this study is to explore the potential association between GIP and the onset of GDM, aiming to provide insights into the complex hormonal interactions that occur during pregnancy. The association of GIP single-nucleotide polymorphisms (SNPs), particularly rs3848460 and rs3895874, was investigated in a cohort of 1,002 Han Chinese individuals to clarify the roles of these genetic variations in GDM. This research presents a novel study on the association between GIP and GDM in an Asian population, which could also be pivotal for advancing our understanding of gestational glucose regulation and have broader implications for metabolic health, ultimately helping to mitigate the long-term health impacts associated with GDM.

## 2 MATERIALS AND METHODS

### 2.1 Study Subjects

For the investigation, 1002 participants were selected, including 500 patients identified with gestational diabetes mellitus (GDM) and 502 healthy pregnant women serving as control subjects. Enrolment criteria were stringent: participants had to provide voluntary written informed consent, be of Han Chinese ethnicity, be 18 years or older, have no history of pregnancy complications, and not be using any glucose-lowering medications during pregnancy (Fig. **1**). Prior to data collection, we conducted a comprehensive literature review to estimate the anticipated effect sizes for the primary outcome variables. Based on existing studies and pilot data, we identified a clinically meaningful effect size that we aimed to detect [21-23]. The investigation was carried out from August 2021 to January 2022 at the Obstetrics Clinic of Shunde Maternal and Child Health Hospital, Guangdong Medical University. Data gathering took place during the gestational period of 24-28 weeks; all participants underwent a routine 75-gram oral glucose tolerance test (OGTT). To initially screen for GDM, pregnant women underwent a 75-gram glucose challenge test. If their blood sugar level one hour after consuming the glucose was below 7.8 mmol/L, the test was considered negative. If the level was 7.8 mmol/L or higher, the test was considered positive. Women with a positive 50-gram glucose challenge test then underwent a more detailed 100-gram oral glucose tolerance test. The diagnosis of GDM adhered to the criteria established by the International Association of Diabetes and Pregnancy Study Groups (IADPSG), encompassing fasting blood glucose (FBG) ≥ 5.1 mmol/L, 1-hour postprandial glucose (PG) ≥ 10.0 mmol/L, or 2-hour PG ≥ 8.5 mmol/L [8, 24]. Patients received treatment in accordance with national guidelines, although this was not the primary focus of the study. The study protocol was previously outlined in detail [25]. Participants who met one or more of these criteria were diagnosed with GDM, whereas those in the control group did not exceed these thresholds. The study received approval from the Ethics Committee and adhered to the principles outlined in the Declaration of Helsinki.

### 2.2 Data Collection

The collected clinical data included age, height, ethnicity, pre-pregnancy weight, parity, glucose levels, and blood pressure. Pre-pregnancy body mass index (pre-BMI) was calculated using the following formula: weight (kg) divided by height squared (m^2^). Obesity classification adhered to Chinese standards, categorizing individuals as normal weight, overweight, underweight, or obese.

### 2.3 Tag SNP Selection and Genotyping

A systematic method was employed for genotyping and selecting tag single-nucleotide polymorphisms (SNPs). The SNPinfo, NCBI-dbSNP, and HapMap databases were used to examine possible single-nucleotide polymorphisms (SNPs) within the GIP gene. The HapMap project, initiated in 2002, focused on SNPs with a minor allele frequency (MAF) greater than 0.05 to detect high-frequency variations with significant special effects and to discover the association between rare mutations and diseases within the population. With a genome-wide association study (GWAS) of Type 2 Diabetes Mellitus (T2DM) and MAF greater than 0.05 in the Asian population, alongside a broad review of recent and relevant literature [26, 27], two candidate SNPs (rs3848460 and rs3895874) associated with GDM risk were observed. During recruitment, two milliliters of fasting peripheral venous blood were collected in an EDTA anticoagulant tube. The samples were then processed, transferred to a 1.5 mL EP tube, and stored at -80°C until further analysis. With the use of the linkage disequilibrium criterion of r^2^≥0.8, SNPs of the GIP gene between 30 kb upstream and 30 kb downstream were selected from the HapMap Phase III JPT+CHB database [28]. The genomic DNA was extracted from cells using the QIAamp DNA Kit provided by Qiagen, Germany. For genotyping, the SNPscan technique developed by Genesky Technologies Inc. in Shanghai, China, was employed. To maintain rigorous quality control, 5% of the samples were randomly selected for duplicate genotyping.

### 2.4 Statistical Analysis

Data analysis was performed using SPSS version 20.0. A *P* -value of less than 0.05 was deemed statistically significant. To assess differences in the genotypic distribution of candidate single-nucleotide polymorphisms (SNPs), allele frequencies, and demographic characteristics between the gestational diabetes mellitus (GDM) group and the control group, independent sample t-tests and chi-square (χ2) tests were conducted. Categorical data were organized and reported as frequencies (n) and percentages (%), while continuous data were expressed as means with standard deviations (¯x ± SD). The Hardy-Weinberg equilibrium (HWE) was evaluated in the control group by using a threshold of *P* >0.05 using Pearson's χ^2^ test [8]. The ones that did not follow the normal distribution were also analyzed using nonparametric tests. Using multivariate logistic regression analysis, we examined the comprehensive relationship between genotypes and GDM across six genetic models: codominant heterozygous, recessive, overdominant, codominant homozygous, allele, and dominant. A one-way analysis of variance (ANOVA) employing the Dunnett-T method, along with post-hoc least significant difference (LSD) tests, was conducted to examine the associations between single-nucleotide polymorphism (SNP) variants and various factors, including glucose levels, neonatal weight, and maternal glucose (MG) concentrations. To assess the linkage disequilibrium (LD) among SNPs within the GIP gene, we utilized the online software tool SHesis, available at http://analysis.bio-x.cn/myAnalysis.php. Variables with lopsided distributions were log^10^-transformed. Logistic regression was used to adjust for potential confounders, and the risk of GDM was assessed by odds ratios (OR) and 95% confidence intervals (CI). Calculations of the frequency distribution excluded haplotypes with a frequency of less than 0.03. GraphPad Prism version 5.01 (GraphPad Software, Inc., San Diego, CA, USA) was used to create statistical graphs, and pre-BMI and age-based subgroup analyses were performed.

## 3 RESULTS

### 3.1 Overview of Clinical Characteristics of Participants

The present study included a total of 1,002 participants, comprising 502 non-GDM controls and 500 cases with gestational diabetes mellitus (GDM), to assess the GIP genotype. Table **1** presents the clinical data, comprising stratified features. The GDM group demonstrated a significantly higher average age of 31.40 ± 4.0 years, in contrast to the control group, which had a lower average age of 29.00 ± 4.0 years (*P* < 0.001). Furthermore, the GDM cases exhibited a marked increase in pre-pregnancy body mass index (BMI) compared to the control group, with values of 20.53 ± 2.58 *versus* 21.51 ± 3.10, respectively (*P* < 0.001). Furthermore, the GDM group displayed elevated levels of diastolic blood pressure (DBP), systolic blood pressure (SBP), fasting plasma glucose (FPG), 1-hour postprandial glucose (1h-PG), and 2-hour postprandial glucose (2h-PG) compared to the control group (*P* < 0.05). These findings shed light on the association between the CDKAL1 genotype and GDM, highlighting the impact of age, pre-BMI, and various metabolic markers on the development of this condition.

### 3.2 SNP Information and HWE Test in the Controls

Table **2** highlights three key SNPs: the minimal allele frequency (MAF), chromosomal location, and the outcomes of the Hardy-Weinberg equilibrium (HWE) analysis in the control group. Adherence to HWE was indicated by a *P* -value greater than 0.05. Our HWE analysis revealed no significant deviation (*P* > 0.05), indicating that the genotype frequencies are in Hardy-Weinberg equilibrium, which is an important quality control measure. The investigation showed MAFs of 0.0662 for rs3848460 and 0.0551 for rs3895874. Furthermore, each SNP's control group adhered to HWE.

### 3.3 Associations between GIP rs3848460, rs3895874 and rs937301 and GDM Risk in All Subjects

A detailed analysis of the association between specific genetic variants (single-nucleotide polymorphisms, or SNPs) of the GIP gene and the risk of gestational diabetes mellitus (GDM) was conducted in a study population. The SNPs analyzed were rs3848460 and rs3895874. Data are presented in terms of genetic models (codominant, allele, dominant, recessive, and overdominant models), comparing cases (n = 500) and controls (n = 502), along with crude and adjusted odds ratios (ORs), confidence intervals (CIs), and *P* -values. Adjustments were made for age, pre-pregnancy body mass index (pre-BMI), systolic blood pressure (SBP), diastolic blood pressure (DBP), and parity.

In the codominant model, GIP rs3848460 showed a significant association with an increased risk of GDM after adjusting for age, pre-BMI, systolic blood pressure (SBP), diastolic blood pressure (DBP), and parity (GG *vs*. AA: OR = 1.717; 95% CI: 1.070-2.754; *P* = 0.025. The recessive model GG *vs* AG+AA suggests an increased risk of GDM (adjusted OR of 1.635), with *P* = 0.034. The GG genotype and G allele demonstrated a statistically significant increased risk with an adjusted OR of 1.760 (*P* = 0.026) and an adjusted OR of 1.250, which is statistically significant (*P* = 0.029), respectively. The GG genotype and G allele were found to be associated with an increased risk of GDM in GIP rs3848460. Conversely, the GA genotype of rs3895874 showed no significant association with the risk of GDM. The risk of GDM was significantly lower in the dominant model (AA+GA *vs*. GG: OR = 0.605; 95% CI: 0.376-0.974; *P* = 0.039) of GIP rs3895874. The AA genotype and A allele were found to be associated with a decreased risk of GDM in GIP rs3895874 after adjustment with an OR of 0.803 (*P* = 0.032). These findings indicated a protective effect against the development of GDM. However, Table **3** indicates no apparent association between GIP rs3895874 and GDM.

### 3.4 Associations between GIP SNPs rs3848460 and rs3895874 and GDM Risk in Subjects Aged Under 30 Years

The study revealed significant associations between specific genotypes of the GIP rs3848460 and rs3895874 polymorphisms and the risk of developing GDM in younger women. We conducted stratified analyses based on age and pre-BMI to further investigate this relationship. The codominant model suggested a protective effect of the AA genotype with a significantly adjusted OR of 0.444, indicating a lower risk of GDM in rs3895874. The allele model supported a reduced risk associated with the A allele, with a significant adjusted OR of 0.738 (*P* = 0.040). In the dominant model, combining the AA and GA genotypes also showed a significantly lower risk of GDM compared to the GG genotype, with an adjusted OR of 0.490 (*P* = 0.045) (Table **4**). The recessive and overdominant models did not demonstrate statistically significant associations.

Analysis revealed a significant association between certain GIP rs3848460 genotypes and an increased risk of GDM in women under 30 years old. In the codominant model, the AG and GG genotypes were associated with higher ORs compared to the AA genotype, indicating a higher risk of GDM with the G allele. The GG genotype had a significantly adjusted OR of 2.323, suggesting a strong association with GDM risk. The allele model confirmed this increased risk associated with the G allele, with a statistically significant adjusted OR of 1.404 (*P* = 0.022).

### 3.5 Associations between GIP rs3848460 and rs3895874 and GDM Risk in Subjects with Pre-BMI of ≥ 24

The study examined the association between specific genetic polymorphisms (GIP rs3848460 and rs3895874) and the risk of developing GDM in individuals with a pre-pregnancy body mass index (BMI) of 24 or higher. On the contrary, the GIP rs3895874 recessive model AA homozygotes had a significantly higher risk of GDM compared to GA and GG carriers combined with an OR of 2.243 (*P* = 0.032), indicating a higher risk of GDM associated with the AA genotype in this polymorphism. Table **5** indicates no significant association between the GIP rs3848460 codominant homozygous model (GG *vs*. AG: OR = 0.312; 95% CI: 0.060-1.635; *P* = 0.168). However, in the allele model, carrying the G allele was associated with a lower risk of GDM compared to the A allele with OR 0.553 (*P* = 0.042), suggesting a potential protective effect of the G allele against GDM.

### 3.6 Association between Polymorphism Genotype and Blood Glucose Level and Neonatal Weight

In the subgroup of individuals younger than 30 years, those with the rs3848460 AA genotype exhibited significantly higher 2-hour glucose levels (*P* < 0.05) compared to carriers of the AG and GG genotypes (Table **6**). Furthermore, individuals with the GG genotype of rs3848460 and rs3895874 in the pre-BMI ≥ 24 subgroup demonstrated significantly elevated 2-hour glucose levels compared to those with the AA and AG genotypes (*P* < 0.05). Additionally, individuals with the GG genotype of rs3848460 and rs3895874 in the pre-BMI ≥ 24 subgroup exhibited higher fasting blood glucose levels (*P* < 0.05) than those with the AA and/or AG genotype (Table **6**). Table **6** also indicates that, in the subgroup of individuals younger than 30 years, the GG genotype of rs3848460 was associated with significantly lower neonatal weight (*P* < 0.05) compared to the AA and AG genotypes. Conversely, the AA genotype of rs3895874 had a greater impact on neonatal weight than the GG genotype (*P* < 0.05) (Table **6**).

In the subgroups of individuals aged < 30 years and those with a pre-pregnancy BMI < 18.5, no statistically significant associations were observed between the genotypes of either SNP and the measured health metrics (FBG, 1 h-PG, 2 h-PG levels, and neonatal weight), as indicated by *p* -values greater than 0.05. Furthermore, the pre-BMI ≥ 24 group showed a statistically significant difference in 2 h-PG levels for certain genotypes of both SNPs, indicating a potential influence of these genetic variants on glucose levels after glucose intake. However, no significant association was found with FBG, 1 h-PG levels, and neonatal weight. The genetic variant GIP rs3848460 showed a significant association with gestational diabetes mellitus (GDM) across all age categories of ≥ 30. However, no significant associations with GDM were found for the genetic variant rs3895874, except in the overdominant model group (GA *vs*. GG+AA: OR = 1.007; 95% CI: 0.704-11.441; *P* = 0.968) (Supplementary Tables **S1** and **S2**). The GA haplotype consisting of these SNPs was significantly associated with higher GDM risk (OR = 1.005; 95% CI: 0.815~1.239; *P* = 0.002). Moreover, haplotype AG was associated with lower GDM risk in subjects aged ≥ 30 years (OR = 0.988; 95% CI: 0.801~1.218; *P* = 0.012) (Supplementary Table **S3**).

### 3.7 Linkage Disequilibrium Analysis

Fig. (**2**) illustrates the linkage disequilibrium (LD) analysis conducted on the candidate single-nucleotide polymorphisms (SNPs) within the GIP gene. The study found that the two GIP SNPs, rs3848460 and rs3895874, were in strong linkage disequilibrium with each other (D′ > 0.99). According to the findings, there was no significant correlation between them and the risk of gestational diabetes mellitus (GDM).

## 4 DISCUSSION

The role of the gastric inhibitory polypeptide (GIP) gene, located on chromosome 17, is increasingly recognized for its involvement in insulin regulation and energy homeostasis, as it encodes an incretin hormone that is critical for these metabolic processes [29]. GIP not only facilitates insulin secretion but also promotes fat storage in adipose tissues, potentially diminishing peripheral insulin sensitivity and impairing glucose tolerance. Studies on mice have shown that the absence of GIP receptors leads to resistance to obesity under high-fat diet conditions, highlighting GIP's role as a promoter of obesity [28, 30, 31]. Additionally, GIP influences glucagon secretion from alpha cells, thereby affecting the body's glucose-regulating capabilities. Its primary secretion from K-cells in the upper intestinal tract and its role in enhancing insulin action by increasing tissue sensitivity to insulin underscore its complex physiological functions [32, 33]. Moreover, emerging research suggests GIP's involvement in inflammatory processes and insulin resistance, expanding its recognized functions beyond traditional incretin activity [34]. The exact role of GIP in GDM development is still under investigation, but it is believed to contribute through several mechanisms. GIP stimulates insulin release from pancreatic beta cells, which can lead to excessive insulin production in pregnant women with underlying insulin resistance.

In this study, involving 1,002 participants, comprising 500 cases of GDM and 502 non-GDM controls, significant clinical differences were observed, particularly in mean age and pre-pregnancy body mass index (BMI), both of which were higher in the GDM group. This supports existing literature that identifies advanced maternal age and higher pre-BMI as risk factors for GDM [35]. The elevated glucose and HbA1c levels found in participants with certain GIP gene variants further confirm the gene's role in glucose metabolism and its potential impact on GDM risk [35]. These findings highlight the multifaceted nature of GDM, which involves a combination of genetic, metabolic, and demographic factors.

Additionally, the observed higher mean age in the GDM group compared to controls in the present study aligns with previous studies [35], suggesting that advanced maternal age is a risk factor for GDM. Maternal age-related changes, such as decreased insulin sensitivity and altered pancreatic function, may predispose older women to GDM. In addition, the significantly elevated pre-BMI in GDM cases underscores the importance of pre-pregnancy BMI as a determinant of GDM risk in the present study. Excessive adiposity before conception can disrupt glucose homeostasis, exacerbating insulin resistance and increasing the likelihood of GDM onset. Future research could explore the interplay between age, pre-BMI, and genetic factors to better understand their combined influence on GDM susceptibility.

The findings from our study highlight two key SNPs, namely rs3848460 and rs3895874, focusing on their minimal allele frequency (MAF), chromosomal location, and adherence to the Hardy-Weinberg equilibrium (HWE) within the control group. To the best of our knowledge, our research represents the first investigation into the association between rs3848460 and rs3895874 and the susceptibility to GDM in the Asian population. Another study [27] also reported that the genotype distributions of the two gene variants in GIP did not deviate significantly from Hardy-Weinberg proportions. The observed MAFs of 0.0662 for rs3848460 and 0.0551 for rs3895874 of the current study suggest that these variants are relatively common within the population under study. A previous study suggests that certain SNPs may influence susceptibility to certain traits or diseases within the population [36, 37].

Furthermore, the findings of this study highlight intriguing associations between genetic variants and GDM, shedding light on potential mechanisms underlying the development of this complex condition. In particular, the examination of six genetic models for single-nucleotide polymorphisms (SNPs) revealed contrasting results, suggesting nuanced relationships between genetic factors and GDM risk.

In the overdominant model, GIP rs3848460 showed a significant association with an increased risk of GDM after adjusting for age, pre-BMI, systolic blood pressure (SBP), diastolic blood pressure (DBP), and parity in the current study. In the present study, the G allele of GIP rs3848460 was significantly associated with GDM after adjustment for confounding factors. These findings were in agreement with the results reported in various previous studies [19, 38].

Additionally, the GIP rs3848460 variant revealed a significant association with GDM in our studies. The presence of the GIP G allele in rs3848460, associated with GDM in the present study, appears to play a role in metabolic disorders linked to chronic inflammatory processes and aging. This was in agreement with a previous study [39]. In addition to its involvement in metabolic disorders, such as impaired postprandial insulin secretion in type 2 diabetes (T2DM) and the pathogenesis of obesity and associated insulin resistance, dysregulated activation of the GIP/GIPR axis has recently been reported in several endocrine tumors, with potential diagnostic and prognostic implications [39]. The association of the AG genotype with increased risk of GDM suggests a potential role in GDM pathogenesis; however, its lack of statistical significance raises questions about its robustness, necessitating replication in larger, independent cohorts.

However, no significant association was observed between GIP rs3895874 and GDM in the overall subjects in the current study. Similar findings were observed in previous studies [27], which stated that no significant association was found between the studied polymorphisms GIP and type 2 diabetes in our study population, and recommended that further studies be conducted. Conversely, the findings for GIP rs3895874 present a paradoxical scenario. While the heterozygous and dominant models suggest a protective effect against GDM, the absence of a significant association in the overall analysis raises questions about the validity of these observations. This inconsistency highlights the complexity of genetic influences on GDM susceptibility and underscores the importance of investigating interactions between genetic variants and environmental factors.

Our investigation, employing six genetic models and stratified analyses based on age and pre-BMI, has unveiled significant associations between specific SNPs and the risk of GDM, particularly among women under 30 years old. One of the noteworthy observations from our study is the significant increase in the incidence of GDM among younger women carrying the GIP rs3895874 recessive genotype (AA), compared to those with the GA or GG genotype. Similar findings were observed in the previous studies [40]. This finding suggests a risk effect conferred by the AA genotype against GDM in this subgroup. However, the study warrants further investigation with larger sample sizes to confirm this association.

Conversely, our analysis also revealed no association between specific GIP rs3848460 genotypes and an increased risk of GDM in women under 30 years old. This suggests a potential susceptibility conferred by certain genetic variants in predisposing younger women to GDM [41, 42]. While some studies have reported associations between specific SNPs and GDM risk, the findings have been inconsistent and often lack replication in independent cohorts [42]. The current study suggests that both genetic factors and environmental influences may contribute to individual susceptibility to GDM.

Likewise, our study investigated the association between genetic variants in the GIP gene and the risk of developing GDM in women with varying pre-pregnancy body mass index (BMI). Our findings revealed contrasting results for two SNPs within the GIP gene, rs3895874 and rs3848460, suggesting a complex interplay between genetic factors and metabolic health during pregnancy. This finding aligns with previous studies that have implicated genetic variations in the GIP gene in the pathogenesis of type 2 diabetes mellitus (T2DM) and obesity-related traits [27]. GIP, also known as glucose-dependent insulinotropic polypeptide, plays a crucial role in glucose homeostasis and insulin secretion. Variations in the GIP gene may influence insulin sensitivity and beta-cell function, thereby affecting an individual's susceptibility to GDM [43].

In contrast, our analysis did not find a significant association between the GIP rs3848460 polymorphism and the risk of GDM, irrespective of the genetic models examined. In other studies as well [27], no association was found between GIP rs3848460 polymorphism and the risk of GDM. This unexpected result suggests that the rs3848460 SNP may not independently influence the risk of GDM in our study population. However, it is important to note that the sample size of our study may have limited the statistical power to detect smaller effect sizes. Further research with larger cohorts is warranted to confirm these findings and explore potential gene-gene or gene-environment interactions that may modulate the impact of GIP variants on the risk of GDM.

Correspondingly, we investigated the association between genetic polymorphisms (specifically, SNPs rs3848460 and rs3895874) with fasting blood glucose (FBG), post-glucose levels (1 h-PG, 2 h-PG), and neonatal weight across different groups categorized by age and pre-pregnancy body mass index (pre-BMI). The study's findings on the non-significant associations between the genotypes of SNPs rs3848460 and rs3895874 and FBG, 1 h-PG, and 2 h-PG levels in most groups (age < 30, pre-BMI < 18.5) align with previous research indicating the complex nature of genetic influences on glucose metabolism [44]. For instance, a study [44] highlighted the polygenic basis of glucose regulation, suggesting that multiple genetic factors and their interactions with environmental factors contribute to the regulation of glucose levels. This complexity could explain why significant associations were not observed in most groups in the study on the association between polymorphisms genotype and blood glucose level and neonatal weight. A notable finding in Table **4** was the significant association between the GG genotype of both SNPs and 2-hour postprandial glucose (2 h-PG) levels in the subgroup with pre-pregnancy BMI of ≥ 24. This finding suggests that genetic influences on glucose metabolism may become more pronounced in the context of higher body mass indices. This observation is supported by a previous research [45], which found that the impact of genetic variants on glucose metabolism is modulated by obesity, indicating a gene-environment interaction effect.

The current study did not find significant associations between the SNPs and neonatal weight, suggesting that these specific genetic variants may not directly influence neonatal outcomes. However, this finding does not rule out the potential impact of other genetic or environmental factors on neonatal weight. A research [46] emphasized the multifactorial determinants of neonatal weight, including maternal health, nutrition, and broader genetic factors, highlighting the need for comprehensive approaches to understand and predict neonatal health outcomes.

Linkage disequilibrium (LD) refers to the non-random association of alleles at different loci. When multiple loci are in LD, alleles at those loci tend to be inherited together more frequently than expected by chance alone. In the context of GIP polymorphisms and GDM risk, LD between rs3848460 A/G and rs3895874 G/A may influence the association between GIP genotypes and GDM risk. Studies investigating the association between GIP polymorphisms and GDM risk have yielded mixed results [27, 19]. Our studies reported that GIP genotypes rs3848460 and rs3895874 were in strong linkage disequilibrium (D′ > 0.99). Some studies have reported significant associations between specific GIP genotypes and an increased risk of GDM, while others have found no significant associations [27, 19]. The presence of LD between multiple loci, such as rs3848460 A/G and rs3895874 G/A, may contribute to these discrepancies by confounding the observed associations.

The LD analysis, along with a substantial sample size, played a crucial role in confirming the credibility of our study. However, it is essential to acknowledge that certain limitations impacted our research findings. Despite this, the association was not observed in either the combined analysis of males and females or in the male population alone. Previous studies have suggested a hormone-dependent association between polymorphisms in genes related to cholesterol metabolism, indicating a potential role for hormones. Associations related to hormones are not a discovery. Therefore, we recommend further research in a different population with a larger sample size to investigate the connection between this variant and LDL cholesterol levels, particularly in female participants. Given that LDL cholesterol, also known as “bad” cholesterol, is a significant risk factor for cardiovascular disease, our findings underscore the need for additional population-based association studies. Functional studies are also necessary to investigate the relationship between GIP and estrogen, abdominal obesity, lipid profile analysis, and environmental factors, in order to gain a deeper understanding of this protein's role in cholesterol metabolism and cardiovascular disease. One of the primary limitations of this study was the inadequate sample size to support a comprehensive analysis of associations. Additionally, the study focused solely on the Chinese population and did not include participants from other genetic backgrounds. It is recommended that future studies obtain a larger sample size for testing.

## CONCLUSION

The findings of the current study suggest that the GIP gene, specifically the rs3848460 polymorphism, is associated with gestational diabetes mellitus (GDM) and may play a role in susceptibility to GDM. Additionally, the study demonstrated the complex genetic basis underlying the development of GDM. The GG genotype and G allele were found to be associated with an increased risk of GDM in GIP rs3848460. However, the GA genotype had no significant association with GDM risk at rs3895874. These findings offer a theoretical foundation for GDM study in order to reduce associated complications for the mother and offspring later in life. To ensure the robustness and generalizability of the present findings, subsequent investigations must employ expanded and heterogeneous subject populations. Furthermore, a critical advancement would be the integration of comprehensive prenatal care data, specifically longitudinal metabolic profiling and lipid profile analysis, to elucidate the intricate interactions between inherited predispositions and environmental influences that contribute to the pathogenesis of GDM.

## Figures and Tables

**Fig. (1):**
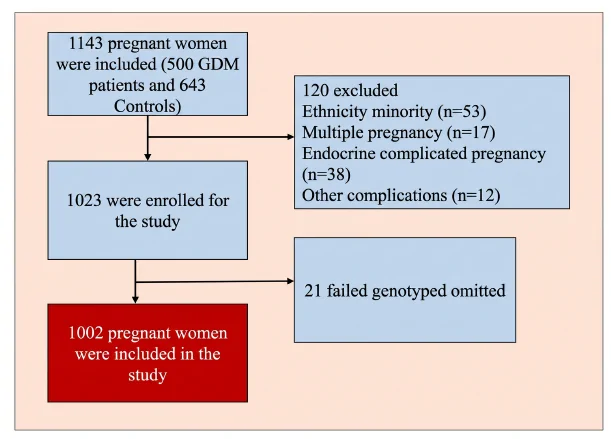
Subject selection criteria for the study focused on Gestational Diabetes Mellitus (GDM).

**Fig. (2):**
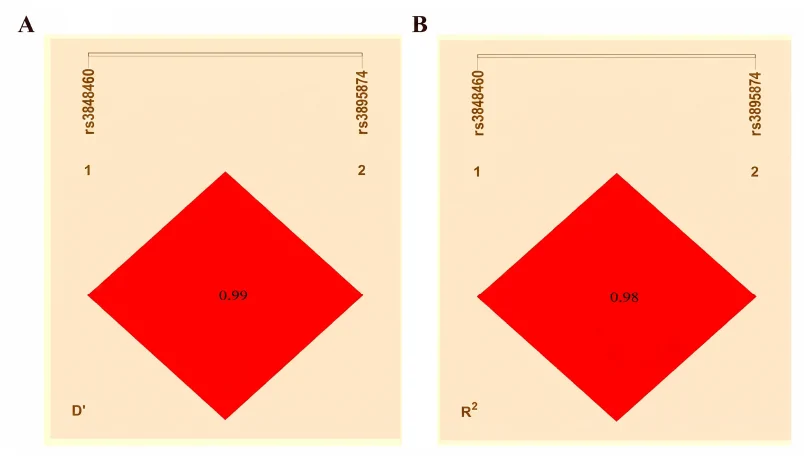
Association between genetic variations (rs7754840 G/C and rs7756992 A/G) within the CDKAL1 gene. (**A**): Linkage disequilibrium (LD) using the D'. (**B**): R^2^ tests.

**Table 1:** Basic and stratified characteristics of participants of the study.

Variables	Healthy Control (502)	GDM Cases (500)	*P*	t/x^2^
**Year, Age**				
<30	26 ± 30	27 ± 20	<0.001	-3.64
≥30	33 ± 20	34 ± 30	0.002	-3.14
**Age, year (mean ± SD)**	29 ± 41	31 ± 42	<0.001	-8.56
**Pre-BMI, kg/m^2^**				
<18.5	17.60 ± 1.50	17.45 ± 0.84	0.45	0.75
18.5 ≤ BMI < 24	20.67 ± 1.41	20.96 ± 1.49	0.009	-2.63
≥24	25.83 ± 3.31	26.16 ± 2.84	0.548	-0.60
** Pre-BMI, kg/m^2^**	20.53 ± 2.58	21.51 ± 3.10	<0.001	-5.42
**Variables among all the data**				
**PG, mmol/L**	4.50 ± 0.31	4.82 ± 0.64	<0.001	-9.75
**1h-PG, mmol/L**	7.66 ± 1.27	10.17 ± 1.60	<0.001	-26.22
**2h-PG, mmol/L**	6.69 ± 0.99	8.91 ± 1.60	<0.001	-25.85
**SBP, mmHg**	114 ± 10	117 ± 11	<0.001	-3.53
**DBP, mmHg**	68 ± 70	70 ± 80	0.001	-3.23
**Parity (n)**				
Primipara	258(51.4)	210(42.1)	0.003	8.88
Multipara	244(48.7)	290(58.0)	-	-

**Note:** Data are presented as mean ± SEM. Diastolic blood pressure =DBP, systolic blood pressure = SBP, fasting plasma glucose = FPG, 1-hour postprandial glucose = 1h-PG, and 2-hour postprandial glucose = 2h-PG. Pre-BMI: pre-gestational body mass index, 1h-PG: 1-hour blood glucose level, 2h-PG: 2-hour blood glucose level. Bold values indicate the *P* < 0.05. 95%

**Table 2:** SNP information and the HWE test in the control groups.

**SNP**	**Min/Maj**	**Chr. position**	**MAF (Control)**	**HWE (*P* )**	**Pearson's *p***	**Fisher's *p***
**rs3848460**	A>G	chr17:48969752	0.0662	0.267	0.874	0.94
**rs3895874**	G>A	chr17:48970506	0.0551	0.145	0.929	0.98

**Abbreviations:** SNP = single-nucleotide polymorphisms, Min = minor allele, Maj = major allele, MAF = frequency of minor allele, HWE = Hardy–Weinberg equilibrium.

**Table 3:** Associations between SNPs in the GIP gene and GDM risk in the overall study population.

SNP	Genetic Model	Cases (n=500)	Control (n=502)	Crude OR (95% CI)	Crude *P*	Adjusted OR (95% CI)	Adjusted *P*
rs3848460	**Codominant Model**						
	AA	243 (48.6)	223 (44.42)	1 (ref)	-	1 (ref)	-
	AG	224 (44.8)	227 (45.22)	1.555 (0.968-2.497)	0.068	1.475 (0.897-2.428)	0.126
	GG	33 (6.6)	52 (10.36)	1.717 (1.070-2.754)	0.025	1.760 (1.070-2.895)	0.026
**Allele Model**							
	A	710(0.71)	673(0.67)	1 (ref)	-	1 (ref)	-
	G	290(0.29)	331(0.329)	0.83 (0.687~1.003)	0.059	1.250 (1.023-1.526)	0.029
**Dominant Model**							
	AA	243 (48.6)	223 (44.42)	1 (ref)	-	1 (ref)	-
	GG+AG	257 (51.4)	279 (55.58)	1.183 (0.923-1.517)	0.185	1.267 (0.974-1.649)	0.078
**Recessive Mode**l							
	AG+AA	467 (93.4)	450 (89.64)	1 (ref)	-	1 (ref)	-
	GG	33 (6.6)	52 (10.36)	1.635 (1.037-2.578)	0.034	1.612 (0.999-2.601)	0.050
**Overdominant Model**							
	AA+GG	276 (55.2)	275 (54.78)	1 (ref)	-	1 (ref)	-
	AG	224 (44.8)	227 (45.22)	1.017 (0.793-1.305)	0.894	1.093 (0.840-1.422)	0.508
rs3895874	**Codominant Model**						
	GG	33 (6.6)	53 (10.56)	1 (ref)	-	1 (ref)	-
	GA	226 (45.2)	226 (45.02)	0.925 (0.714-1.199)	0.557	0.856 (0.651-1.127)	0.268
	AA	241 (48.2)	223 (44.42)	0.576 (0.360-0.923)	0.022	0.560 (0.341-0.919)	0.022
**Allele Model**							
	G	292(0.292)	332(0.33)	1 (ref)	-	1 (ref)	-
	A	708(0.708)	672(0.669)	0.834 [0.69~1.008]	0.066	0.803 (0.658-0.981)	0.032
**Dominant Model**							
	GG	33 (6.6)	53 (10.56)	1 (ref)	-	1 (ref)	-
	AA+GA	467 (93.4)	449 (89.44)	0.599 (0.380-0.942)	0.027	0.605 (0.376-0.974)	0.039
**Recessive Model**							
	GA+GG	259 (51.8)	279 (55.58)	1 (ref)	-	1 (ref)	-
	AA	241 (48.2)	223 (44.42)	0.859 (0.670-1.101)	0.231	0.801 (0.616-1.142)	0.099
**Overdominant Model**							
	GG+AA	274 (54.8)	276 (54.98)	1 (ref)	-	1 (ref)	-
	GA	226 (45.2)	226 (45.02)	0.993 (0.774-1.273)	0.954	1.066 (0.820-1.387)	0.633

**Note:** Adjusted *P* value calculated by logistic regression with adjustment for age, pre-BMI, SBP, DBP, and parity. Bold values indicate *P* < 0.05.

**Table 4:** The associations between GIP rs3848460 and rs3895874 and GDM risk in subjects aged < 30.

	Genetic Model	Cases (n=192)	Control (n=304)	Crude OR (95% CI)	Crude *P*	Adjusted OR (95% CI)	Adjusted *P*
rs3848460	**Codominant Model**						
	AA	96 (50)	133 (43.75)	1 (ref)	-	1 (ref)	-
	AG	83 (43.23)	136 (44.74)	1.643 (0.822-3.285)	0.160	1.790 (0.867-3.699)	0.116
	GG	13 (6.77)	35 (11.51)	1.943 (0.976-3.869)	0.059	2.323 (1.125-4.793)	0.041
**Allele Model**							
	A	270 .71.6)	402 (66.1)	1 (ref)	-	1 (ref)	-
	G	109 (28.3)	206 (33.8)	1.293 (0.979-1.708)	1.708	1.404 (1.050-1.876)	0.022
**Dominant Model**							
	AA	96 (50)	133 (43.75)	1 (ref)	-	1 (ref)	-
	GG+AG	96 (50)	171 (56.25)	1.286 (0.895-1.847)	0.174	1.428 (0.976-2.088)	0.066
**Recessive Model**							
	AG+AA	179 (93.23)	269 (88.49)	1 (ref)	-	1 (ref)	-
	GG	13 (6.77)	35 (11.51)	1.792 (0.922-3.480)	0.085	2.040 (1.016-4.093)	0.045
**Overdominant Model**							
	AA+GG	109 (56.77)	168 (55.26)	1 (ref)	-	1 (ref)	-
	AG	83 (43.23)	136 (44.74)	1.063 (0.739-1.530)	0.742	1.130 (0.773-1.652)	0.528
rs3895874	**Codominant Model**						
	GG	13 (6.77)	35 (11.51)	1 (ref)	-	1 (ref)	-
	GA	85 (44.27)	135 (44.41)	0.898 (0.615-1.310)	0.576	0.822 (0.554-1.222)	0.333
	AA	94 (48.96)	134 (44.08)	0.529 (0.266-1.055)	0.071	0.444 (0.215-0.917)	0.028
**Allele Model**							
	G	111 (28.9)	205 (33.7)	1 (ref)	-	1 (ref)	-
	A	273 (7.1)	(403 (66.2)	0.799 (0.606-1.055)	0.113	0.738 (0.553-0.986)	0.040
**Dominant Model**							
	GG	13 (6.77)	35 (11.51)	1 (ref)	-	1 (ref)	-
	AA+GA	179 (93.23)	269 (88.49)	0.558 (0.287-1.084)	0.085	0.490 (0.244-0.984)	0.045
**Recessive Model**							
	GA+GG	98 (51.04)	170 (55.92)	1 (ref)	-	1 (ref)	-
	AA	94 (48.96)	134 (44.08)	0.822 (0.572-1.181)	0.288	0.744 (0.509-1.087)	0.126
**Overdominant Model**							
	GG+AA	107 (55.73)	169 (55.59)	1 (ref)	-	1 (ref)	-
	GA	85 (44.27)	135 (44.41)	1.006 (0.699-1.447)	0.976	1.064 (0.728-1.554)	0.748

**Note:** Adjusted *P* value calculated by logistic regression with adjustment for age, pre-BMI, SBP, DBP, and parity. Bold values indicate *P* < 0.05.

**Table 5:** The associations between GIP rs3848460 and rs3895874 and GDM risk in subjects with pre-BMI of ≥ 24.

	Genetic Model	Cases (n=97)	Control (n=42)	Crude OR (95% CI)	Crude *P*	Adjusted OR (95% CI)	Adjusted *P*
rs3848460	**Codominant Model**						
	AA	35 (36.08)	23 (54.76)	1 (ref)	-	1 (ref)	-
	AG	51 (52.58)	17 (40.48)	0.545 (0.110-2.711)	0.459	0.548 (0.104-2.885)	0.478
	GG	11 (11.34)	2 (4.76)	0.277 (0.056-1.365)	0.115	0.312 (0.060-1.635)	0.168
**Allele Model**							
	A	121 (62.3)	63 (7.5)	1 (ref)	-	1 (ref)	-
	G	73 (37.6)	21 (2.5)	0.553 (0.312-0.980)	0.042	0.603 (0.331-1.097)	0.098
**Dominant Model**							
	AA	35 (36.08)	23 (54.76)	1 (ref)	-	1 (ref)	-
	GG+AG	62 (63.92)	19 (45.24)	0.466 (0.224-0.973)	0.042	0.523 (0.239-1.146)	0.105
**Recessive Model**							
	AG+AA	86 (88.66)	40 (95.24)	1 (ref)	-	1 (ref)	-
	GG	11 (11.34)	2 (4.76)	0.391 (0.083-1.847)	0.236	0.414 (0.083-2.076)	0.285
**Overdominant Model**							
	AA+GG	46 (47.42)	25 (59.52)	1 (ref)	-	1 (ref)	-
	AG	51 (52.58)	17 (40.48)	0.613 (0.294-1.278)	0.192	0.678 (0.309-1.486)	0.332
rs3895874	**Codominant Model**						
	GG	11 (11.34)	2 (4.76)	1 (ref)	-	1 (ref)	-
	GA	52 (53.61)	17 (40.48)	2.069 (0.966-4.431)	0.061	1.874 (0.835-4.206)	0.128
	AA	34 (35.05)	23 (54.76)	3.721 (0.754-18.370)	0.107	3.332 (0.635-17.479)	0.155
**Allele Model**							
	G	74 (38.1)	21 (2.5)	1 (ref)	-	1 (ref)	-
	A	120 (61.8)	63 (7.5)	0.541 (0.305-0.958)	0.035	0.584 (0.321-1.062)	0.078
**Dominant Model**							
	GG	11 (11.34)	2 (4.76)	1 (ref)	-	1 (ref)	-
	AA+GA	86 (88.66)	40 (95.24)	2.558 (0.542-12.084)	0.2558	2.405 (0.482-12.007)	0.285
**Recessive Model**							
	GA+GG	63 (64.95)	19 (45.24)	1 (ref)	-	1 (ref)	-
	AA	34 (35.05)	23 (54.76)	2.243 (1.073-4.687)	0.032	2.030 (0.929-4.437)	0.076
**Overdominant Model**							
	GG+AA	45 (46.39)	25 (59.52)	1 (ref)	-	1 (ref)	-
	GA	52 (53.61)	17 (40.48)	0.588 (0.282-1.226)	0.157	0.640 (0.293-1.396)	0.262

**Note:** Adjusted *P* value calculated by logistic regression with adjustment for age and SBP. Bold values indicate *P* < 0.05.

**Table 6:** Association between polymorphism genotype and blood glucose level and neonatal weight.

Groups	SNP	Genotype	FBG (mmol/L)	1 h-PG (mmol/L)	2 h-PG (mmol/L)	Neonatal Weight (g)
Age < 30	rs3848460	AA	4.72 ± 0.06	8.59 ± 0.15	7.56 ± 0.13	3186.51 ± 24.49
		AG	4.55 ± 0.02	8.42 ± 0.12	7.21 ± 0.10	3170.62 ± 23.02
		GG	4.55 ± 0.07	8.03 ± 0.28	7.45 ± 0.20	3169.17 ± 58.80
		*F*	0.253	0.637	0.074	0.888
		*P*	> 0.05	> 0.05	> 0.05	> 0.05
	rs3895874	GG	4.55 ± 0.07	8.03 ± 0.28	7.45 ± 0.20	3169.17 ± 58.80
		GA	4.56 ± 0.02	8.44 ± 0.12	7.21 ± 0.10	3174.98 ± 23.00
		AA	4.71 ± 0.06	8.58 ± 0.15	7.56 ± 0.13	3182.37 ± 24.53
		*F*	0.992	0760	0.907	0.994
		*P*	> 0.05	> 0.05	> 0.05	> 0.05
Pre-BMI < 18.5	rs3848460	AA	4.49 ± 0.10	8.30 ± 0.26	7.26 ± 0.22	3169.25 ± 50.64
		AG	4.50 ± 0.05	8.07 ± 0.30	7.31 ± 0.22	3088.91 ± 46.13
		GG	4.51 ± 0.16	7.59 ± 0.45	7.10 ± 0.30	2981.25 ± 118.06
		F	0.999	0.648	0.962	0.479
		*P*	> 0.05	> 0.05	> 0.05	> 0.05
	rs3895874	GG	4.51 ± 0.16	7.59 ± 0.45	7.10 ± 0.30	2981.25 ± 118.06
		GA	4.50 ± 0.05	8.07 ± 0.30	7.31 ± 0.22	3088.91 ± 46.13
		AA	4.49 ± 0.10	8.30 ± 0.26	7.26 ± 0.22	3169.25 ± 50.64
		*F*	0.996	0.823	0.940	0.692
		*P*	> 0.05	> 0.05	> 0.05	> 0.05
Pre-BMI ≥ 24	rs3848460	AA	4.98 ± 0.13	9.40 ± 0.52	7.56 ± 0.39^a^	3248.75 ± 79.86
		AG	4.72 ± 0.10	9.18 ± 0.29	7.56 ± 0.27^a^	3280.00 ± 65.16
		GG	4.98 ± 0.14	10.01 ± 0.86	9.33 ± 0.23^b^	3420.00 ± 240.36
		*F*	1.000	0.929	0.063	0.958
		*P*	> 0.05	> 0.05	< 0.05	> 0.05
	rs3895874	GG	4.98 ± 0.14	10.01 ± 0.86	9.33 ± 0.23^b^	3420.00 ± 240.36
		GA	4.76 ± 0.10	9.16 ± 0.28	7.52 ± 0.26^a^	3295.42 ± 64.27
		AA	4.95 ± 0.14	9.43 ± 0.54	7.60 ± 0.41^a^	3231.30 ± 81.40
		*F*	0.706	0.668	0.055	0.758
		*P*	> 0.05	> 0.05	< 0.05	> 0.05

**Abbreviations:** SNP: single-nucleotide polymorphisms; a,b,c *p* -value<0.05 indicates statistical significance.

## Data Availability

The data presented in this research are deposited in the EVA repository, with the project accession number PRJEB71647.

## LIST OF ABBREVIATIONS

GIP: Glucose-dependent Insulinotropic Polypeptide
GDM: Gestational Diabetes Mellitus
T1DM: Type 1 diabetes
T2DM: Type 2 diabetes
GIPRs: GIP Receptors
BMI: Body Mass Index
SNPs: Single-nucleotide Polymorphisms
OGTT: Oral Glucose Tolerance Test
IADPSG: International Association of Diabetes and Pregnancy Study Groups
MAF: Minor Allele Frequency
LSD: Least Significant Difference
HWE: Hardy-Weinberg Equilibrium

## STANDARDS OF REPORTING

STROBE guidelines were followed.
