# Supplementary material for: Association of Glucose-Dependent Insulinotropic Polypeptide Receptor Polymorphisms rs3848460 and rs3895874 with the Risk of Gestational Diabetes Mellitus
Source: Endocr Metab Immune Disord Drug Targets. 2025 Aug 5;26:E18715303367483. doi: 10.2174/0118715303367483250728105845 (PMC13631867; doi:10.2174/0118715303367483250728105845)
Supplement: Supplementary file 1 [file EMIDDT-26-E18715303367483_SD1.pdf]

Supplementary Material

Association of Glucose-Dependent Insulinotropic Polypeptide Receptor Polymorphisms rs3848460 and rs3895874 with the Risk of Gestational Diabetes Mellitus

Gyan Watson Ray[1,2,3], Hengli Zhang[1,2,3], Taotao Shao[1,2], Taili Yang[3], Yue Wei[3], Mianqin Li[4], Xiaoqun Che[5,6,*], Qiaoli Zeng[1,2,3,*] and Runmin Guo[1,2,3,*]

[1]*Department of Internal Medicine, Shunde Women and Children's Hospital (Maternity and Child Healthcare Hospital of Shunde Foshan), Guangdong Medical University, Foshan, Guangdong, China;* [2]*Key Laboratory of Research in Maternal and Child Medicine and Birth Defects, Guangdong Medical University, Foshan, Guangdong, China;* [3]*Maternal and Child Research Institute, Shunde Women and Children's Hospital (Maternity and Child Healthcare Hospital of Shunde Foshan), Guangdong Medical University, Foshan, Guangdong, China;* [4]*Department of Ultrasound, Shunde Women and Children's Hospital (Maternity and Child Healthcare Hospital of Shunde Foshan), Guangdong Medical University, Foshan, Guangdong, China;* [5]*Department of Obstetrics, Shunde Women and Children's Hospital (Maternity and Child Healthcare Hospital of Shunde Foshan), Guangdong Medical University, Foshan, Guangdong, China;* [6]*Reproductive Medicine Center, Shunde Women and Children's Hospital (Maternity and Child Healthcare Hospital of Shunde Foshan), Guangdong Medical University, Foshan, Guangdong, China*

Table S1. The associations between GIP rs3848460, and rs3895874 and GDM risk in age ≥ 30 subjects.

SNP	Genetic Model	Cases (n=308)	Control (n=198)	Crude OR (95 % CI)	Crude *P*	Adjusted OR (95 % CI)	Adjusted *P*
rs3848460	**Codominant Model**						
	AA	147 (47.73)	90 (45.45)	1 (ref)		1 (ref)	
	AG	141 (45.78)	91 (45.96)	1.317 (0.655-2.647)	0.440	1.254 (0.613-2.566)	0.536
	GG	20 (6.49)	17 (8.59)	1.388 (0.691-2.789)	0.357	1.387 (0.678-2.839)	0.371
**Allele Model**							
	A	435 (70.60)	271(68.40)	1 (ref)		1 (ref)	
	G	181(29.30)	125(31.50)	0.902 [0.686~1.186]	0.544	1.131 (0.854-1.498)	0.391
**Dominant Model**							
	AA	147 (47.73)	90 (45.45)	1 (ref)		1 (ref)	
	GG+AG	161 (52.27)	108 (54.55)	1.096 (0.766-1.567)	0.617	1.142 (0.790-1.651)	0.480

**Recessive Model**							
	AG+AA	288 (93.51)	181 (91.41)	1 (ref)		1 (ref)	
	GG	20 (6.49)	17 (8.59)	1.352 (0.690-2.650)	0.379	1.319 (0.662-2.630)	0.431
**Overdominant Model**							
	AA+GG	167 (54.22)	107 (54.04)	1 (ref)		1 (ref)	
	AG	141 (45.78)	91 (45.96)	1.007 (0.704-1.441)	0.968	1.057 (0.731-1.527)	0.770
rs3895874	**Codominant Model**						
	GG	20 (6.49)	18 (9.09)	1 (ref)		1 (ref)	
	GA	141 (45.78)	91 (45.96)	0.938 (0.646-1.362)	0.737	0.890 (0.607-1.305)	0.550
	AA	147 (47.73)	89 (44.95)	0.673 (0.338-1.340)	0.259	0.673 (0.332-1.366)	0.273
**Allele Model**							
	G	127(0.32)	181(0.293)	1 (ref)		1 (ref)	
	A	269(0.679)	435(0.706)	0.881 [0.67~1.158]	0.822	0.906 (0.747-1.099)	0.316
**Dominant Model**							
	GG	20 (6.49)	18 (9.09)	1 (ref)		1 (ref)	
	AA+GA	288 (93.51)	180 (90.91)	0.694 (0.358-1.348)	0.281	0.713 (0.361-1.409)	0.331
**Recessive Model**							
	GA+GG	161 (52.27)	109 (55.05)	1 (ref)		1 (ref)	
	AA	147 (47.73)	89 (44.95)	0.894 (0.625-1.280)	0.541	0.854 (0.591-1.235)	0.403

**Overdominant Model**							
	GG+AA	167 (54.22)	107 (54.04)	1 (ref)		1 (ref)	
	GA	141 (45.78)	91 (45.96)	1.007 (0.704-11.441)	0.968	1.062 (0.734-1.535)	0.750

Note: Adjusted *P* value calculated by logistic regression with adjustment for age.

Table S2 . The associations between GIP rs3848460, and rs3895874 and GDM risk in pre-BMI < 18.5 subjects.

	Genetic Model	Cases (n=67)	Control (n=95)	Crude OR (95 % CI)	Crude *P*	Adjusted OR (95 % CI)	Adjusted *P*
rs3848460	**Codominant Model**						
	AA	36.00 (53.73)	44.00 (46.32)	1 (ref)		1 (ref)	
	AG	26.00 (38.81)	42.00 (44.21)	1.114 (0.336-3.691)	0.859	1.205 (0.359-4.168)	0.768
	GG	5.00 (7.46)	9.00 (9.47)	1.473 (0.453-4.786)	0.520	1.573 (0.463-5.341)	0.468
**Allele Model**							
	A	98 (73.1)	130 (68.4)	1 (ref)		1 (ref)	
	G	36 (26.8)	60 (31.5)	1.256 (0.770-2.050)	0.361	1.272 (0.770-2.102)	0.348
**Dominant Model**							
	AA	36.00 (53.73)	44.00 (46.32)	1 (ref)		1 (ref)	
	GG+AG	31.00	51.00	1.346 (0.719-	0.353	1.346 (0.707-	0.365

		(46.27)	(53.68)	2.520)		2.562)	
**Recessive Model**							
	AG+AA	62.00 (92.54)	86.00 (90.53)	1 (ref)		1 (ref)	
	GG	5.00 (7.46)	9.00 (9.47)	1.298 (0.415-4.061)	0.654	1.394 (0.427-4.554)	0.583
**Overdominant Model**							
	AA+GG	41.00 (61.19)	53.00 (55.79)	1 (ref)		1 (ref)	
	AG	26.00 (38.81)	42.00 (44.21)	1.250 (0.661-2.362)	0.493	1.223 (0.637-2.349)	0.545
rs3895874	**Codominant Model**						
	GG	5.00 (7.46)	9.00 (9.47)	1 (ref)		1 (ref)	
	GA	26.00 (38.81)	42.00 (44.21)	0.757 (0.392-1.461)	0.406	0.766 (0.390-1.504)	0.439
	AA	36.00 (53.73)	44.00 (46.32)	0.679 (0.209-2.207)	0.520	0.636 (0.187-2.158)	0.468
**Allele Model**							

	G	36 (26.8)	60 (31.5)	1 (ref)		1 (ref)	
	A	98 (73.1)	130 (68.3)	0.796 (0.488-1.298)	0.361	0.786 (1.061-1.194)	0.348
**Dominant Model**							
	GG	5.00 (7.46)	9.00 (9.47)	1 (ref)		1 (ref)	
	AA+GA	62.00 (92.54)	86.00 (90.53)	0.771 (0.246-2.412)	0.654	0.717 (0.220-2.344)	0.583
Recessive Model							
	GA+GG	31.00 (46.27)	51.00 (53.68)	1 (ref)		1 (ref)	
	AA	36.00 (53.73)	44.00 (46.32)	0.743 (0.397-1.391)	0.353	0743 (0.390-1.414)	0.365
**Overdominant Model**							
	GG+AA	41.00 (61.19)	53.00 (55.79)	1 (ref)		1 (ref)	
	GA	26.00 (38.81)	42.00 (44.21)	1.250 (0.661-2.362)	0.493	1.223 (0.637-2.349)	0.545

Note: Adjusted *P* value calculated by logistic regression with adjustment for age.

Table S3. Haplotype analysis of the rs3848460 and rs3895874 SNPs of the GIP gene for the GDM and controls.

Haplotype	Case(freq)	Control(freq)	Chi^2 *P*	OR [95% CI]
AG	224(0.224)	227(0.226)	0.012	0.988 [0.801~1.218]
GA	226(0.226)	226(0.225)	0.002	1.005 [0.815~1.239]
AA	484(0.484)	446(0.444)	3.187	1.173 [0.984~1.398]
GG	66(0.066)	105(0.104)	9.555	0.605 [0.438~0.834]
